# Experimental Investigation of Spray Drying Breakup Regimes of a PVP-VA 64 Solution Using High-Speed Imaging †

**DOI:** 10.3390/pharmaceutics16121547

**Published:** 2024-12-02

**Authors:** Cooper Welch, Mobaris Khawar, Benjamin Böhm, Andreas Gryczke, Florian Ries

**Affiliations:** 1 Technical University of Darmstadt, Department of Mechanical Engineering, Reactive Flows and Diagnostics, Otto-Berndt-Str. 3, 64287 Darmstadt, Germany; welch@rsm.tu-darmstadt.de (C.W.); mobaris.khawar@hotmail.com (M.K.); boehm@rsm.tu-darmstadt.de (B.B.); 2 AbbVie Deutschland GmbH & Co. KG, Knollstraße, 67061 Ludwigshafen am Rhein, Germany; andreas.gryczke@abbvie.com

**Keywords:** spray drying, breakup regimes, PVP-VA, amorphous solid dispersions, Ohnesorge diagram, diffuse back-illumination

## Abstract

**Background:** Atomization plays a key role in spray drying, a process widely used in the pharmaceutical, chemical, biological, and food and beverage industries. In the pharmaceutical industry, spray drying is particularly important in the preparation of amorphous solid dispersions, which enhance the bioavailability of active pharmaceutical ingredients when mixed with a polymer. **Methods:** In this study, a 3D-printed adaptation of a commercial spray dryer nozzle (PHARMA-SD^®^ PSD-1, GEA Group AG) was used to investigate the atomization of PVP-VA 64 polymer solutions under varying flow conditions using high-speed diffuse back-illumination. **Results:** Unlike pure water, the atomization process of the polymer solution was governed by viscous effects rather than surface tension, as indicated by stringing effects in the liquid core and the formation of larger droplets. In addition, the classical Ohnesorge diagram accurately predicted the atomization regime with increasing Reynolds numbers and could be modified to reasonably predict the breakup regime by considering the transitions between regime boundaries. **Conclusions:** The use of such a modified diagram facilitates the efficient selection of viscous fluid solutions and process parameters to achieve complete spray formation.

## 1. Introduction

Newly discovered active pharmaceutical ingredients (APIs) often have very poor water solubility, which is associated with low oral bioavailability—the ability to be absorbed by the body's cells. This problem affects approximately 60–70% of drugs currently in development [1,2]. Recent developments in the pharmaceutical industry have enabled various techniques to formulate such APIs. A widely used method for increasing the bioavailability of APIs is amorphization and dispersion on a molecular-level carrier, where the crystalline lattice of the substance is broken down into an amorphous state that can be better absorbed by the body [3]. To stabilize the amorphous state, APIs are typically dispersed to a molecular level in a polymer matrix, resulting in an amorphous solid dispersion (ASD). 

The manufacture of ASDs can be categorized into two basic methodological groups: solvent-based methods and melting/fusion-based methods [4], the former of which is relevant to this work. In the last decade, the FDA approved 48 new ASD-based products [5]. With 27 of the 48 products using spray drying, it is the most widely used process for the manufacture of ASD drug intermediates. This is due to its favorable scalability from the laboratory to the industrial scale and its applicability for heat-sensitive APIs, among other reasons [4]. Spray drying is a well-established technique in the manufacturing process of powders, not only in the pharmaceutical industry, but also in the food, chemical, ceramic, and polymer industries [6]. In the context of the production of ASDs in the pharmaceutical industry, spray drying is used to rapidly produce particles by atomizing and rapidly evaporating a liquid feed solution consisting of the API, a polymer carrier, and a solvent, such as acetone, with a hot gas. Several process variables are critical to controlling the atomization process, and thus, the particle properties: the inlet and outlet temperature, ambient humidity and pressure, and flow rate of the evaporation gas and liquid feed; the nozzle design, especially the diameter; and the solid content of the solution [4]. In addition, formulation variables such as the API composition; solid content in the feed, for example, insoluble and soluble materials and their ability to absorb and retain solvent molecules; viscosity; surface tension; and solvent type and composition play important roles in the output of the process [4]. Due to the complex interplay of the process and formulation variables, improving the spray drying process and product quality is likely to remain a research focus for years to come.

Spray atomization is a well-established research topic in many industries, particularly for fuel injection applications in the transportation sector. In recent decades, improvements in high-speed (HS) imaging technology have enabled the high-resolution measurements of sprays in time and 2D and 3D space, an improvement in spray visualization over the popular 0D and 1D techniques of the past [7]. In particular, diffuse back-illumination (DBI) is a commonly used technique for uniform imaging of sprays and is particularly useful for determining spray boundaries in post-processing [8]. In experimental setups for HS DBI, a diffuse light source—typically a pulsed laser or LED—and an HS camera are placed on either side of the spray, and the spray boundary is determined later after several post-processing steps, such as image dewarping and background correction. In addition to experimental methods, spray formation has also been characterized using computational fluid dynamics, as reported in [9,10,11,12] or elsewhere. These studies primarily concentrate on the characterization of fuel injection processes in combustion engines.

Although spray drying is a widely used technique in the pharmaceutical industry, few researchers have studied it with realistic solution formulations using HS optical diagnostics. In particular, the atomization behavior of non-Newtonian fluids in two-fluid nozzle sprays is largely unknown. A study employing backlit imaging of the spray of non-Newtonian juice fiber suspensions using a two-fluid nozzle showed that the viscoelastic fiber suspensions formed filamentary structures and consequently larger droplets than those of Newtonian fluids [13]. Furthermore, the study showed that increasing the gas–liquid mass flow ratio (GLR) resulted in better atomization in both Newtonian and non-Newtonian fluid suspensions. Closer to the present work, Poozesh et al. used HS shadowgraph imaging and a PIV algorithm to investigate the effects of process and formulation variables on the atomization properties of pharmaceutical-relevant solutions of a 5% *w*/*w* polymer carrier [14]. By using non-dimensional parameters to classify the atomization process, Poozesh et al. related the parameters specific to spray behavior phenomena, such as the liquid breakup length and the spray angle. 

The present study aims to extend the work of Poozesh et al. [14] by using HS DBI to investigate the spray atomization behavior of a 3D-printed adaptation of a commercial two-fluid spray dryer nozzle with non-Newtonian solutions of a 20% *w*/*w* polymer. In addition, this paper extends previous work by the same authors [15] by including a complete test matrix of various materials and presenting correlation functions to describe the predicted behavior of spray drying atomization characteristics. This paper is presented as follows: First, the experimental methodology is presented to describe the materials used, test stand, imaging setup, and digital image processing techniques. Then, the results are presented to show a characteristic comparison of the spray morphology and the resulting differences of the breakup regimes with different process and formulation variables. Finally, the results are summarized, conclusions are drawn, and recommendations for future work are given.

## 2. Materials and Methods

### 2.1. Materials

Solutions of copovidone (PVP-VA 64) and distilled water were prepared outside the experimental test bench and mixed using a pitched blade turbine mixing impeller (R 1345, IKA-Werke GmbH & Co. KG, Staufen im Breisgau, Germany). Due to safety concerns in the open laboratory environment, water was used as a solvent instead of the commonly used acetone. Therefore, sodium dodecyl sulfate (SDS) was added to samples of each solution to reduce the surface tension σ, allowing a wider range of material properties to simulate various viscosities and surface tensions that are closer to those of acetone (*σ*_acetone_ = 0.023 N/m [16]). It is important to note that, as discussed by Porfirio et al. [17], the impact of the polymer on surface tension is negligible; therefore, σ of the solvent system can be considered representative of the polymeric solutions. Values of σ for water–SDS solutions can be obtained from [18] or similar works. The values of the dynamic viscosity μ for PVP-VA 64 in water at 25 °C were taken from [19], which used a capillary viscosimeter and did not provide measurement standard deviations. In PVP-VA 64–water solutions, the viscosity can vary for a number of reasons, including polymer manufacturing variability, the measuring instrument, and the solution temperature. As the uncertainties of these mixtures are not quantified, it should be noted that they were prepared from the same batch of PVP-VA 64 in the same facility and the measurements were each made on the same day in the same room temperature environment. Any uncertainties in the material properties should be similarly reflected in the calculation of the Ohnesorge number and should, therefore, be insignificant to the results of this study. In this study, 0%, 10% (see Appendix A), and 20% polymer solutions were used to cover a wide range of viscosities. The properties of the solutions are shown in Table 1, and further properties are provided in Appendix A.

### 2.2. Two-Fluid Spray Dryer Nozzle and Test Bench

**Figure 1:**
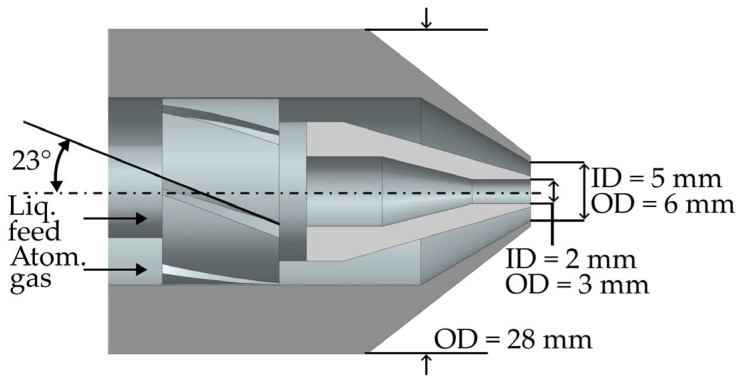
Cross-sectional view of the two-fluid spray dryer nozzle tip.

The test bench used in this study was specially designed to simulate the spray drying process in a simplified open laboratory configuration. To accomplish this, a spray dryer nozzle was placed inside an open aluminum profile cage. The two-fluid nozzle used in this study was adapted from a 3D-scanned commercial spray dryer commonly used on an industrial scale (PHARMA-SD^®^ PSD-1, GEA Group AG, Düsseldorf, Germany). The original nozzle is a good manufacturing practice (GMP) device and, therefore, cannot be removed from its GMP facility. In addition, the adapted nozzle was shortened by approximately half to a length of 225 mm to reduce the space needed in the laboratory. This reduction in the length did not affect the development of the gas and liquid flows at the exit of the nozzle. The adapted nozzle used in this study was printed using a stereolithography 3D printer, and minor adjustments were made by filing off excess roughness. Figure 1 shows a cross-sectional view of the nozzle geometry. In this nozzle assembly, the liquid was fed through the center core nozzle (inner diameter (ID) of 2 mm, outer diameter (OD) of 3 mm) and atomized by gas that was accelerated and swirled by swirl fins before flowing coaxially around the liquid jet (ID of 5 mm, OD of 6 mm).

The liquid feed was supplied using a custom-made syringe pump consisting of a 500 mL syringe mounted on a frame and compressed by a drive-screw turned by a stepper motor (Tinkerforge GmbH, Schloß Holte-Stukenbrock, Germany). In order to convert the mass flow rate of the liquid feed from the volume flow rate, a density of 1000 kg/m^3^ was assumed for all solutions since the values were within 5% from one another (see Appendix A for the sensitivity of the assumed solution density on results). The atomization gas used was dry air (relative humidity of 1.8%), which was precisely controlled by a newly calibrated digital mass flow controller (accuracy: ±0.5%, EL-FLOW^®^ Select F-202AV, Bronkhorst High-Tech B.V., Ruurlo, The Netherlands). For mass flow rates lower than 2 kg/h, the mass flow controller was replaced with a lower range model (accuracy: ±0.5% of reading, EL FLOW^®^ Prestige FG-201CV, Bronkhorst High-Tech B.V.). The characterization measurements of the spray dryer were conducted with an air co-flow of 75 m^3^/h supplied by a high-range mass flow controller (accuracy: ±1% of reading, IN-FLOW ‘High-Flow’ F-206AI, Bronkhorst High-Tech B.V.) through a custom air rectifier consisting of 4 inlets, grids, a bed of beads, and honeycomb channels. In the breakup regime analysis, no co-flow was used to reduce the effects of surrounding flow on the liquid breakup. Furthermore, all air used in this experiment was room temperature at approximately 20 °C. The stepper motor and mass flow controller were controlled using LabVIEW version 2022 Q3 (National Instruments Corporation, Austin, TX, USA). The nozzle was placed vertically facing down 1 m above an optical table and surrounded by curtains that protected the optical components. Above the nozzle, a 1 m × 1 m exhaust hood was used for gas extraction and to enclose the nozzle assembly. Since the primary atomization took place in the near-field of the nozzle tip, it was assumed that the flow was not affected by the wall boundary of the table.

### 2.3. Optical Setup

Diffuse back-illumination was achieved by placing a high-power pulsed LED (LPS III, ILA_5150 GmbH, Berlin, Germany) and a 12-bit HS CMOS camera (HSS6, LaVision GmbH, Göttingen, Germany) on either side of the nozzle outside of the curtained test bench. Windows of approximately 15 cm × 15 cm were cut out to allow optical access into the curtained enclosure. The light from the LED was shaped with a plano-convex lens (f=100 mm) and a Fresnel lens (f=152.4 mm). In addition, an engineered diffuser with a 30° divergence angle was placed inside the enclosure to homogenize the light. To protect the diffuser from soiling, an air curtain with several small nozzles was placed between the diffuser and the spray dryer nozzle. 

**Figure 2:**
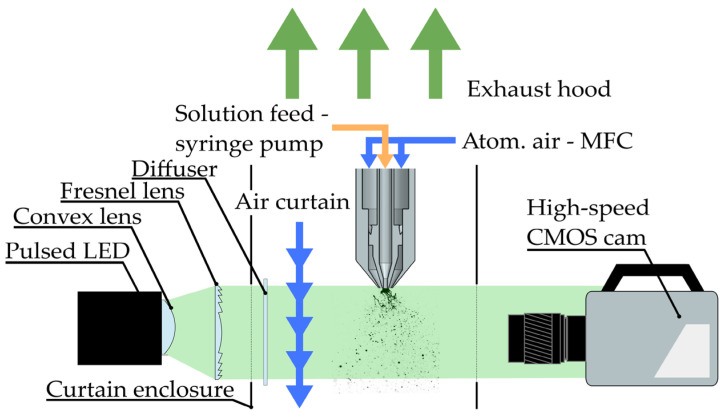
Schematic of the experimental setup. The placement of objects in the schematic is not to scale.

The camera was set up approximately 1.5 m from the nozzle and equipped with a Zeiss (Oberkochen, Germany) Makro-Planar T* 2/100 lens, with the aperture set to *f*/8. For each measurement, 5000 images were acquired at 500 Hz, and an air blower bulb was used to prevent any stray particles from reaching the lens. The relatively low acquisition rate was justified in order to collect more statistics in the 512 px × 512 px field of view. The LED was set to 90% intensity and synchronized with the camera’s shutter by an HSCv2 (LaVision GmbH) controller. The pulse duration was set to 25 µs, effectively setting the exposure time to 25 µs. Figure 2 shows an unscaled schematic of the experimental test bench and optical components. The optical resolution was measured using a Siemens star. It was calculated to be 204 µm (4.90 lpmm) in the focal plane and 303 µm (3.30 lpmm) in the plane 10 mm out of focus. The resolutions were calculated using a 10% threshold criterion of the modulation transfer function (MTF) curve.

### 2.4. Data Processing and Evaluation

Digital image processing was performed to gain insight from the 12-bit raw images. First, the raw images were dewarped and placed in real-world coordinates using a 3rd-order polynomial correction (DaVis version 8.4, LaVision GmbH, Göttingen, Germany) from images of a custom backlit rectangular target under the nozzle. Next, the perspective-corrected images were subjected to a background correction by subtracting the mean of 20 images just before a spray event from the spray images to correct for intensity inhomogeneities. Finally, the intensity of the corrected images was inverted so that the spray areas had high intensity counts, and the resulting images were either normalized to the maximum intensity of the spray from an experimental run and analyzed directly or binarized for further quantitative analysis. 

**Figure 3:**
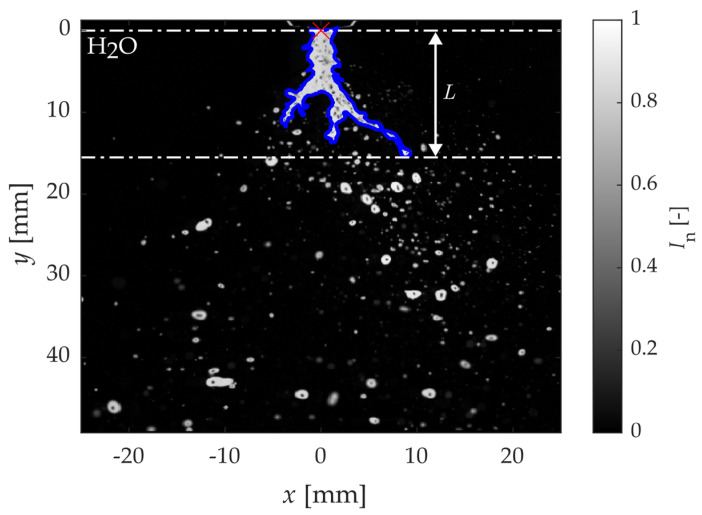
Example spray image and resulting binarization (blue outline). The liquid breakup length *L* is represented by the white arrow.

The binarization process involved several steps to distinguish the liquid jet and the breakup from the atomized spray. First, the images were normalized by the maximum intensity of all images in a single experimental run. Then, a threshold of 50% of the maximum normalized intensity *I*_n_ was used as the initial binarization. Next, all but the largest connected structure was removed, and the holes within the structure were filled. Finally, the liquid breakup length *L* was obtained by recording the maximum distance along the injector axis of a binarized pixel from the injector tip. An example spray image of water with the extracted liquid boundary and *L* is shown in Figure 3. The blue outline represents the binarized outline of the liquid jet, and the red × represents the location of the injector tip. The parameters (H_2_O with 8 kg/h liquid feed and 2 kg/h atomization gas) of the example spray show borderline atomization. They were chosen to clearly outline the liquid boundary of the spray and represent a comparison case between different solution formulations.

Processing was conducted and figures were generated in MATLAB version 2022b (The MathWorks, Inc., Natick, MA, USA). Some figures, such as the experimental setup, and some figure post-processing were made using Inkscape version 1.2.

## 3. Results and Discussion

### 3.1. Primary Atomization Characteristics

**Figure 4:**
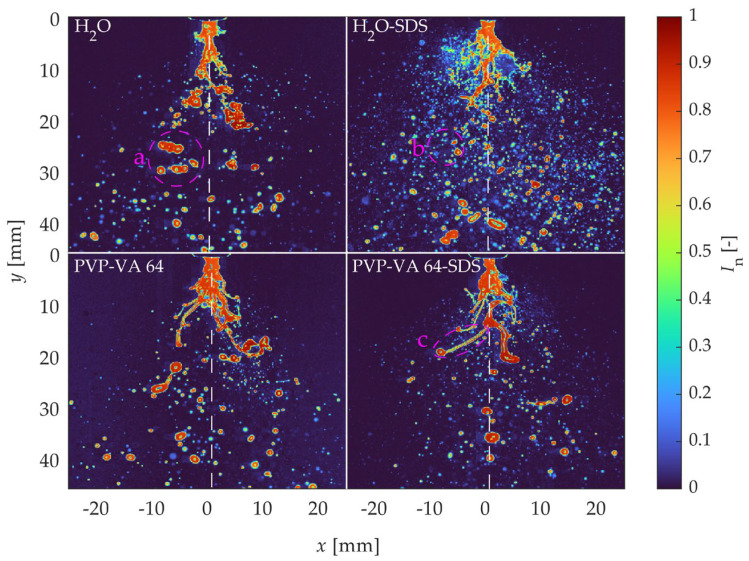
Comparison of instantaneous primary breakup images of liquid feed of 8 kg/h and atomization gas flow rate of 2 kg/h. White symmetry lines are placed with respect to the nozzle tip, and phenomena are highlighted by magenta ellipses: (a) representative larger droplet clusters; (b) representative smaller droplets in and out of focus; (c) representative long, stringy filaments.

Before investigating the breakup regimes of the different solutions, the behavior of the primary breakup had to first be characterized. Figure 4 shows a comparison of the instantaneous normalized spray images with the liquid feed rate of 8 kg/h and the atomization gas flow of 2 kg/h. With the selected low GLR, which represented a borderline primary atomization regime, the differences in the features of the liquid jets and the primary breakup of the varying formulation parameters were more pronounced. From left to right, the surface tension decreased, and from top to bottom, and the viscosity increased. In comparison to the low-surface-tension case, normal H_2_O (also displayed in Figure 3) generally showed larger droplets and a thicker liquid core (see cluster “a” highlighted in magenta for representative droplets). In contrast, the SDS case showed many smaller droplets in and out of focus (see cluster “b” highlighted in magenta for representative droplets), and the liquid core appeared to break up closer to the injector tip. Similar to the comparison of higher and lower surface tension for the water cases, the normal PVP-VA 64 case had a thicker liquid core, especially visible at the nozzle tip, and fewer small droplets than its SDS counterpart. However, at high viscosities, the breakup process was dominated by the viscosity rather than the surface tension. While some similarities existed between the two base fluid samples, the polymer suspensions generally had longer liquid core lengths than the water solutions (see filament “c” highlighted in magenta for representative phenomenon). This was due to the higher viscosity of the 20% PVP VA 64 solutions, which exhibited filaments that strung together longer liquid structures.

**Figure 5:**
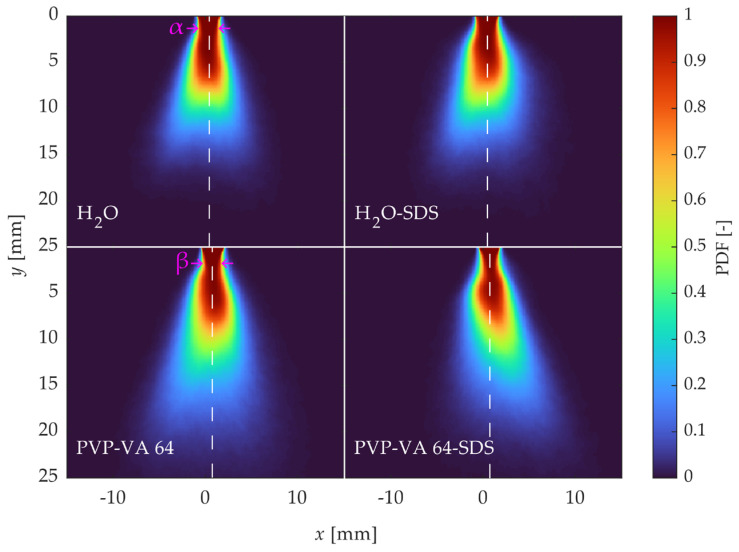
Comparison of binarized PDFs for liquid feed of 8 kg/h and atomization gas flow rate of 2 kg/h. White symmetry lines are placed with respect to the nozzle tip, and phenomena are highlighted by magenta arrows to display the differences in the liquid core width.

**Figure 6:**
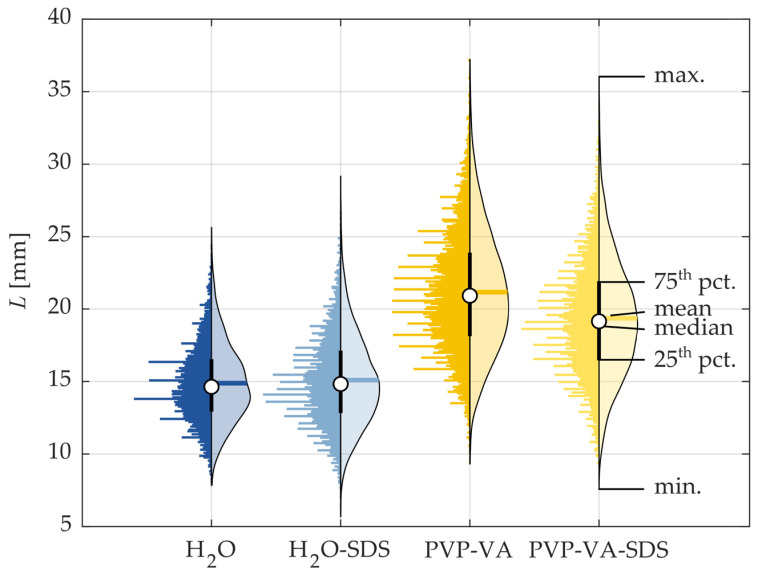
Comparison of violin plots of *L* for liquid feed of 8 kg/h and atomization gas flow rate of 2 kg/h.

Rather than relying on characteristic individual images to differentiate the atomization of the different solutions that were arbitrarily selected, a statistical approach provided a quantitative comparison. Figure 5 and Figure 6 show statistical comparisons of the sprays using the same liquid feed rate and atomization air flow rate as in Figure 4. Figure 5 displays the probability density function (PDF) of the binarized liquid breakup regime, where the average of all binarized images was considered, yielding a spatial probability map where a value of 1 indicates that all images had a liquid core there. Several observations can be made from the PDF comparisons. First, the width of the liquid core varied, especially between the low-viscosity (water) and high-viscosity (polymer solution) samples; the PDFs of the water samples showed wider liquid cores before the samples interacted with the atomization air (see features α and β highlighted in magenta for a representative comparison). This can be attributed to the greater likelihood of the more viscous solutions being stretched and pulled by the force of the air. In addition, the PDFs of the SDS solutions displayed a high asymmetry compared to those of their higher-surface-tension counterparts, indicating an exaggerated effect of the rotational flow on the liquid breakup when the solutions had a lower surface tension. This effect is likely due to the tendency of fluids with higher surface tension to adhere to solid interfaces (here, the nozzle). 

A quantitative comparison of the liquid breakup length *L* is shown in Figure 6 using violin plots. The PDFs show that the PVP-VA 64 solutions had greater liquid penetration lengths; however, the violin plots, which show the mean (thick colored horizontal line), the median (white circle), the interquartile range (thick black vertical line), the histogram distribution of all data (left side of violin, normalized to have equal width for each sample), and the kernel density estimation (KDE) of the distribution (right side of violin), show that the polymer solutions also had a wider distribution of *L*. This indicates that the stringy filaments characteristic of higher viscosity resulted in 42.9% and 29.1% increases in the *L* for the normal and SDS cases, respectively. Each solution had almost a normal distribution; however, there were more extremes toward the maxima of the *L* rather than the minimum due to the constraint of the injector tip on the minimum side. Interestingly, while there was a clear difference in the distribution for the normal PVP-VA 64 case versus its SDS counterpart, the water cases had approximately the same mean and median *L*, while the distribution for the SDS case was slightly more spread out. Following this comparison of the primary liquid breakup of the different solutions under the same operating constraints, the next section compares the breakup regimes and their predictability using the classical Ohnesorge diagram.

### 3.2. Spray Breakup Regimes

**Figure 7:**
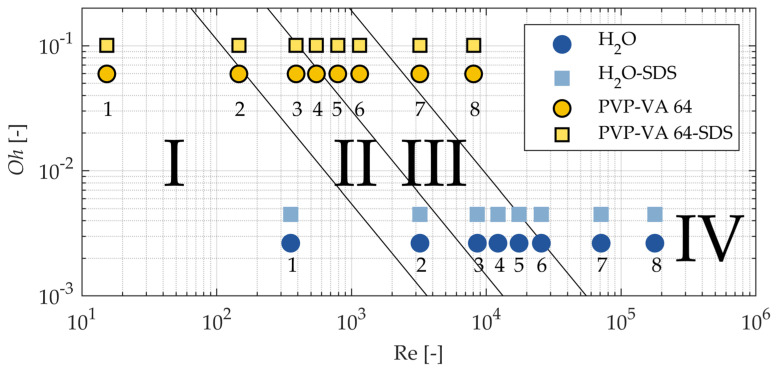
Ohnesorge breakup regime diagram and conditions for spray images considered in Figure 8 and Figure 9. The labelled regimes are as follows: I: Rayleigh regime, II: first wind-induced regime, III: second wind-induced regime, and IV: atomization regime [21].

In fluid dynamics, dimensionless numbers are useful when generalizing the phenomena of different conditions and geometries. In the context of spray atomization, Wolfgang von Ohnesorge first proposed a method to predict the liquid breakup regime of jets based on a dimensionless number that compares viscous and surface tension forces. Ohnesorge plotted this dimensionless number, later named the Ohnesorge number *Oh*, against the Reynolds number, Re, of several fluids under different conditions and developed the so-called Ohnesorge diagram to predict the breakup regimes of jets with prescribed properties and conditions [20]. The Ohnesorge and Reynolds numbers can be described in a two-fluid nozzle by the following equations [14]:

(1) Re=vg−vlDlρlμ

and

(2) $$\mathrm{Oh}=\frac{\mu }{\sqrt{\rho_{l}\sigma D_{l}}}$$

where *v* is the velocity of the gas g and liquid l phases, respectively, *D*_l_ is the nozzle orifice diameter, and *ρ* is the density. Accordingly, four breakup regimes have been defined by subsequent researchers: I: Rayleigh regime, II: first wind-induced regime, III: second wind-induced regime, and IV: atomization regime [21]. Since the Ohnesorge diagram is typically used in automotive injector configurations, the present study aimed to test its applicability to a two-fluid nozzle with different liquid solutions. Figure 7 displays an adaptation of the Ohnesorge diagram with the conditions for the four breakup regimes shown in [21]. Eight operating parameters with increasing atomization gas velocity were tested for each liquid solution and are shown in Figure 7. Each formulation had a characteristic dripping or straight liquid jet condition where the atomization gas was turned off (labeled “1”) and a characteristic high atomization condition (labeled “8”).

**Figure 8:**
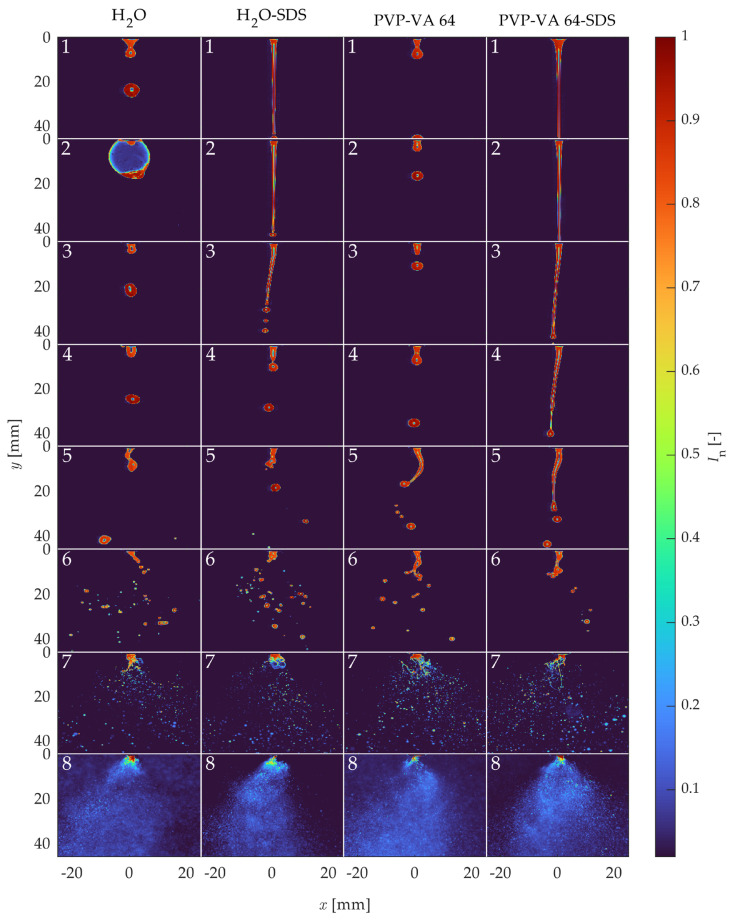
Instantaneous spray breakup images of the conditions numbered 1 through 8 in Figure 7.

Normalized instantaneous images for each representative point on Figure 7 are shown in Figure 8. Before analyzing the breakup regimes, some interesting phenomena should first be discussed. The most obvious phenomenon was the bubble that appeared in the H_2_O case at point 2. This bubble occurred only in this case due to an instability caused by the high surface tension, which caused the water to stick to the nozzle tip, combined with the low flow rate of atomization air. This bubbling occurred continuously, as a new bubble formed each time a large bubble burst. The next notable phenomenon occurred at point 7 of the PVP-VA 64-SDS case. In this condition, the engineered diffuser was soiled due to the large amount of atomized polymer solution in contact with it. This fouling resulted in a glare point in the recorded images that appeared near the center of the field of view. Finally, in the high-atomization cases at point 8, with the exception of the water with SDS solution, the high number of atomized droplets obscured the line-of-sight, which appeared as a higher intensity background. This occurred despite the background subtraction processing step because there were too many droplets moving upward toward the exhaust vent. While these phenomena were largely due to the experimental parameters, they were independent of the regime analysis and could, therefore, be ignored.

Several other observations can be made by examining the images of the regimes. As the Re increased, the steady flow of liquid droplets (high surface tension) or jets (low surface tension) became more unstable. At point 3, which is between the prescribed first and second wind-induced regimes in the Ohnesorge diagram, the instability in the SDS cases became apparent as the liquid jets began to angle to the left and form droplets closer to the nozzle tip. Next, at point 5, the high-surface-tension droplet cases also showed instability, characteristic of first wind-induced breakup, with the high-viscosity PVP-VA 64 solutions showing longer, stringier jets prior to droplet formation. Then, true second wind-induced breakup began to occur for each condition at point 6, with a significant shortening of the liquid core and many more small droplets appearing. Finally, the two atomization cases at points 7 and 8 showed obvious atomization, while the high-atomization case at point 8 displayed almost no visible liquid jet.

**Figure 9:**
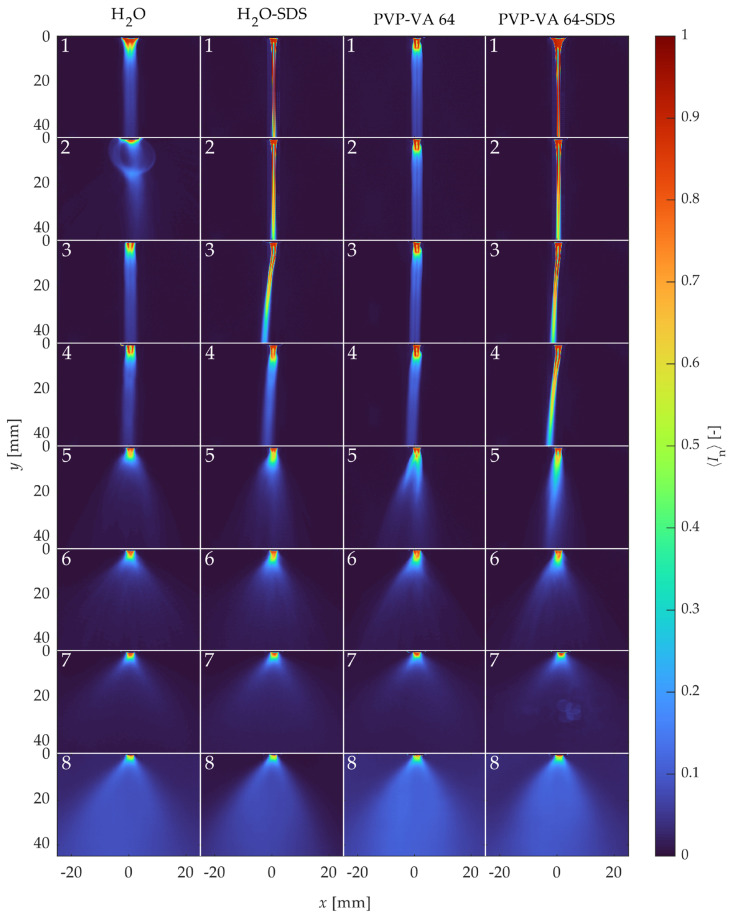
Mean spray breakup images of the conditions numbered 1 through 8 in Figure 7.

Each classical breakup regime from the Ohnesorge diagram for all solutions are shown in Figure 8, although mainly with shifts from the expected regime location. However, the instantaneous images were chosen arbitrarily and may not reflect the operating point as a whole, especially in the low-Re cases. Therefore, Figure 9 shows the mean of the breakup regimes over all the images. The bubbling for point 2 of the water, the glare effect of case 7 of PVP-VA 64-SDS, and the high-intensity background for point 8 all appear prominently in the mean images. However, the mean images also indicate further differences. For example, the single shot images of the droplets (high-surface-tension solutions) show no obvious instability due to the atomization flow at operating point 3, the mean images show a deviation from a perfect downward jet (compared to point 1). In addition, since the one-sided jets always went to the left, it is likely that they were also directed perpendicular to the measurement plane due to the clockwise motion of the atomization air. Finally, the atomization cases of operating points 7 and 8 did not have noticeably different average spray characteristics despite the significant differences in their liquid feed properties and the presence of larger droplets in the lower atomization air point.

**Figure 10:**
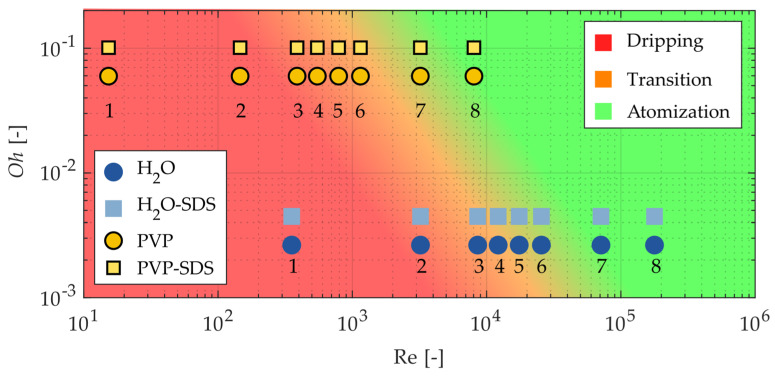
Modified Ohnesorge diagram for the two-fluid spray dryer nozzle of this study.

The similar average spray characteristics for points 7 and 8, coupled with the fact that these atomization operating points closely followed their predicted results based on the Ohnesorge diagram, indicate that the Ohnesorge diagram can be used to reasonably predict atomization even in the presence of highly viscous, non-Newtonian fluids. However, as in all cases shown, any points that border a regime on the Ohnesorge diagram, such as operating point 6, may not accurately reflect the outcome. Therefore, a modified Ohnesorge diagram is proposed in Figure 10, where the color represents the breakup regime. Since there was no real cut-off in the observed regimes, a gradual transition from one regime to the next is more realistic and should be considered when predicting the liquid jet breakup regimes of two-fluid nozzles. Using such a modified diagram, the characteristics of the resulting atomization can be predicted; for example, if larger droplets are desired, an operating point near the orange–green transition should be selected.

## 4. Conclusions

This paper presents an experimental investigation of the atomization characteristics of a widely used commercial two-fluid nozzle spray dryer for four different liquid solutions: water, water with surfactant (sodium dodecyl sulfate, SDS), 20% copovidone, and 20% copovidone with surfactant. High-speed diffuse back-illumination imaging was used to compare the differences attributed to the four solutions. The low-surface-tension water solution was characterized by violent breakup and a smaller liquid core with a higher number of smaller droplets than the normal water fluid. For the copovidone (PVP-VA 64) solutions, however, the breakup was dominated by the effects of the high viscosity, rather than the surface tension, and long, stringy filaments branched from the liquid core before separating into large droplets. The longer liquid core of the high-viscosity cases is evident from the increased mean and median liquid breakup lengths.

Analysis of the four liquid solutions under eight different operating parameters of increasing gas–liquid mass flow ratios was performed to test the feasibility of predicting the liquid jet breakup regimes of the two-fluid nozzle using the classical Ohnesorge diagram. Although breakup characteristics cannot be precisely predicted near the regime boundaries, a proposed Ohnesorge diagram is useful for estimating breakup regimes with a smooth transition from regime to regime rather than strict cut-off criteria. Two operating points within the atomization regime were correctly predicted, with a more modest atomization condition closer to the transition between second wind-induced breakup and atomization. 

In future work, further analysis of atomization-related properties can be performed to further improve the prediction of the properties of the resulting sprays. By quantifying droplet properties, such as size distribution, under different operating conditions and solution parameters, the atomization regime of the modified Ohnesorge diagram can be further improved to include possible atomization property outcomes. In addition, by examining the available data, which include more fluid solutions and operating parameters, correlation functions can be developed to predict the liquid breakup length and spray angle.

## Figures and Tables

**Table 1:** Properties of solution formulations.

Property	H_2_O	H_2_O-SDS	PVP-VA 64	PVP-VA 64-SDS
% Water (*w*/*w*)	100.0	99.80	80.00	79.84
% Polymer (*w*/*w*)	0.00	0.00	20.00	19.96
% Surfactant (*w*/*w*)	0.00	0.20	0.00	0.20
Dyn. viscosity μ (Pa-s)	0.001	0.001	0.023	0.023
Surface tension σ (N/m)	0.072	0.025	0.072	0.025
Density ρ (kg/m^3^)	997	997	1037	1037

**Figure A1:**
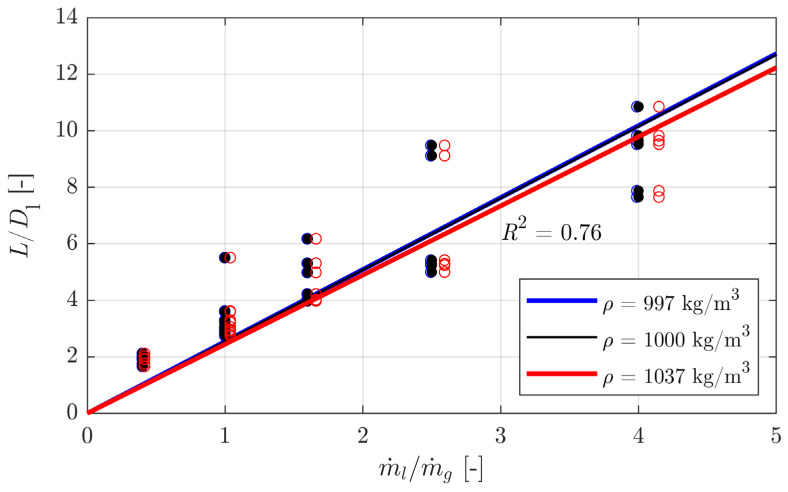
Sensitivity of liquid-to-gas mass flow ratio to solution density, and its effect on the linear fit of the liquid breakup length *L*. The circles represent the measured data while the bold lines represent the linear fits.

**Figure A2:**
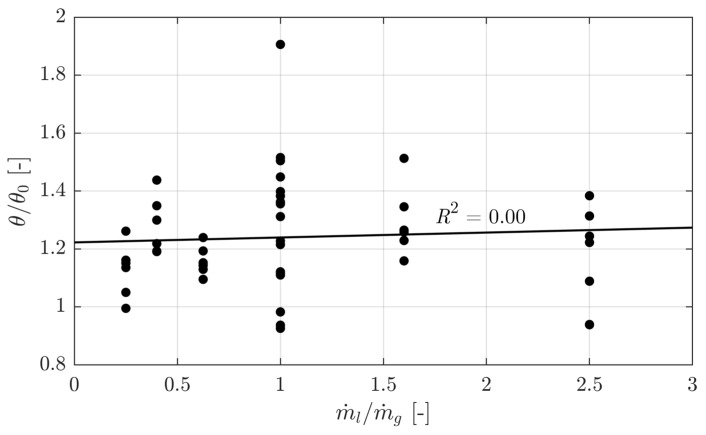
Ratio of spray angle to nozzle opening angle versus liquid-to-gas mass flow ratio. The dots represent the measured data while the bold line represents the linear fit.

## Data Availability

The original contributions presented in the study are included in the article, interested parties can contact the corresponding author for data availability.

## Appendix A. Correlation Functions for Liquid Breakup Length and Spray Angle

The data presented in the main body of this paper are part of a larger experimental matrix for the determination of correlation functions used to predict liquid breakup length *L* and spray angle *θ*. The experimental matrix consists of six different solutions, each with nine operating parameters, for a total of 54 operating conditions. Table A1 lists the physical properties of the six solutions, and Table A2 lists the nine operating parameters that make up the complete test matrix.

**Table A1:** Physical properties of all solutions used to determine the correlation functions.

Solution	1	2	3	4	5	6
% Water (*w*/*w*)	100.0	99.80	90.00	89.90	80.00	79.84
% Polymer (*w*/*w*)	0.00	0.00	10.00	9.90	20.00	19.96
% Surfactant (*w*/*w*)	0.00	0.20	0.00	0.20	0.00	0.20
Dyn. viscosity μ (Pa-s)	0.001	0.001	0.009	0.009	0.023	0.023
Surface tension σ (N/m)	0.072	0.025	0.072	0.025	0.072	0.025
Density ρ (kg/m^3^)	997	997	1017	1017	1037	1037

**Table A2:** Operating parameters used to determine the correlation functions.

Parameter	1	2	3	4	5	6	7	8	9
Liquid feed ${\dot{m}}_{l}$ (kg/h)	2	2	2	5	5	5	8	8	8
Atom. flow rate ${\dot{m}}_{g}$ (kg/h)	2	5	8	2	5	8	2	5	8

As in the main body of this paper, the liquid feed mass flow rates assumed a solution density of 1000 kg/m^3^ instead of those shown in Table A1. This assumption had a minimal effect on the final correlation functions, as illustrated in Figure A1, which compares the fitted curves of the liquid breakup length *L* divided by the nozzle diameter *D*_l_ to the liquid-to-gas mass flow ratio with different solution densities. The effect of density was greatest at higher liquid-to-gas mass flow ratios; however, these conditions were less relevant for the spray drying because complete atomization was not achieved. In addition, for the analysis of *L*, conditions with an atomization flow rate of 8 kg/h were removed because there was no liquid core at such high gas flow rates. The test matrix of the 36 remaining operating conditions resulted in a linear relationship between the liquid breakup length *L* and the mass flow ratio. Since there was no spray or liquid penetration when the solution mass flow rate was zero, the linear regression fits were assumed to have a y-intercept of zero, with a coefficient of determination *R*^2^ of 0.76, independent of the assumed solution density.

Based on the linear fit of the data assuming ρ=1000 kg/m^3^, as shown in Figure A1, a correlation function relating *L* to the operating conditions can be expressed by the following equation:

(A1) $$\frac{L}{D_{l}}=2.54\frac{{\dot{V}}_{l}}{{\dot{V}}_{g}}\frac{\rho_{l}}{\rho_{g}}.$$

Similar to the liquid breakup length *L*, the spray angle *θ* is an important quantity in shaping the morphology of atomized sprays. In this work, *θ* was calculated from the mean spray image of each operating condition by extracting the radial position where the intensity *I* reached 50% of the maximum intensity of the image at each pixel along the injector axis. A linear fit of the radial position along the axis was then performed to extract the spray boundary on both the left and right sides of the image, which could be used together to provide the total *θ* from the injector tip to the position where the coefficient of determination *R*^2^ equaled 0.995. Due to a lack of atomization when the mass flow rates for the solution feed and atomizing gas were 8 kg/h and 2 kg/h, respectively, this condition was removed from the test matrix, leaving the analysis to 48 operating conditions. Figure A2 shows the ratio of the calculated spray angle to the nozzle opening angle *θ*_0_ versus the liquid-to-gas mass flow ratio and the resulting linear fit. As indicated by the *R*^2^ of 0.00, the linear fit is almost perfectly horizontal. Most of the data points fall within an angle ratio range of 0.8 to 1.6, with a mean and standard deviation of 1.24 and 0.18, respectively. As with turbulent round jets, the opening angle of this spray drying nozzle appeared to be independent of the mass flow rate. This means that the spray angle for this nozzle can be predicted relatively well based solely on the geometry of the nozzle and is independent of the mass flow ratio; thus, the spray angle can be approximated as 1.2 times the opening angle of the nozzle, as follows:

(A2) $$\theta \;=1.2\theta_{0}$$
